# Development of dextran nanoparticles for stabilizing delicate proteins

**DOI:** 10.1186/1556-276X-8-197

**Published:** 2013-04-27

**Authors:** Fei Wu, Zhihua Zhou, Jing Su, Liangming Wei, Weien Yuan, Tuo Jin

**Affiliations:** 1 School of Pharmacy, Shanghai Jiao Tong University, 800 Dongchuan Road, Shanghai 200240, China; 2 State Key Laboratory of Electronic Thin Film and Integrated Devices, School of Microelectronics and Solid-state Electronics, University of Electronic Science and Technology of China, Chengdu 610054, China; 3 Key Laboratory for Thin Film and Microfabrication of the Ministry of Education, Institute of Micro/Nano Science and Technology, Shanghai Jiao Tong University, Shanghai 200240, China

**Keywords:** Dextran nanoparticles, Protein, Aggregation, Bioactivity, Acidic microenvironment

## Abstract

One of the most challenging problems in the development of protein pharmaceuticals is to deal with stabilities of proteins due to its complicated structures. This study aims to develop a novel approach to stabilize and encapsulate proteins into dextran nanoparticles without contacting the interface between the aqueous phase and the organic phase. The bovine serum albumin, granulocyte-macrophage colony-stimulating factor (GM-CSF), granulocyte colony-stimulating factor (G-CSF), *β*-galactosidase, and myoglobin were selected as model proteins. The proteins were added into an aqueous solution containing the dextran and polyethylene glycol, and then encapsulated into dextran nanoparticles by aqueous-aqueous freezing-induced phase separation. The encapsulation efficiency and recovery of dextran nanoparticles were determined. The dextran nanoparticles loaded with proteins were characterized by scanning electron microscopy and particle size analysis. The protein aggregation was determined by size-exclusion chromatography-high-performance chromatography, and the bioactivity of proteins recovered during formulation steps was determined. The bioactivity of GM-CSF, G-CSF, and *β*-galactosidase were examined by the proliferation of TF-1 cell, NSF-60 cell, and ortho-nitrophenyl-*β*-galactoside assay, respectively. The results of bioactivity recovered show that this novel dextran nanoparticle can preserve the protein's bioactivity during the preparation process. LysoSensor™ Yellow/Blue dextran, a pH-sensitive indicator with fluorescence excited at two channels, was encapsulated into dextran nanoparticles to investigate the ability of dextran nanoparticles to resist the acidic microenvironment (pH < 2.5). The result shows that the dextran nanoparticles attenuate the acidic microenvironment in the poly (lactic-co-glycolic acid) microsphere by means of the dilution effect. These novel dextran nanoparticles provided an appealing approach to stabilize the delicate proteins for administration.

## Background

Proteins play crucial roles in virtual pharmaceutical science covering cytokine, antibody, enzyme, supplements, and vaccine [1-5]. Considerable progress in the molecular biology and genetic engineering during the past 3 decades has led to a significant increase in the number of approved protein drugs covering nearly 150 diseases [6]. Protein has several advantages over small molecule drugs [7]. However, proteins are prone to denaturation and degradation, owning to their flexible structure which brought forward several formidable challenges in the process of formulation, storage, and *in vivo* release [8-10]. It is now well known that compared with peptide drug, protein has a complex structure which poses the formidable challenges involved in formulating nanoparticles loaded with proteins [11-13]. The traditional water-in-oil-in-water double emulsion is a common approach to prepare nanoparticles loaded with proteins. However, the interface between the aqueous phase and the organic phase is a major disadvantage of the traditional emulsion method, and has been identified as a major cause of protein denaturation and aggregation [14]. The formation of the interface between the aqueous phase and the organic phase is a common destabilizing reason for proteins and generally results in interfacial adsorption followed by protein unfolding and aggregation [15-17]. In order to protect the interface between the aqueous phase and the organic phase, some metal ions, such as calcium, magnesium, and zinc, were used as protein stabilizers because they bind to a protein and make the overall protein structure more rigid, compact, and stable [18,19]. The effect of stabilization depends on the concentration of mental ions and the type of proteins used. Moreover, the effect of metal ions on protein stability can be significantly influenced by the negative counter ions. A well-known method to form fine protein particles is the precipitation of protein via bivalent metal ions [20,21]. A complex of proteins with bivalent metal ions in an aqueous phase was found as an effective way to form protein particles, such as human growth hormone, but this method was also reported to facilitate aggregation when applied to some proteins such as erythropoietin [22,23]. The aggregation of the protein can result in an immune response. Especially, protein aggregates could increase the immunogenicity of various therapeutic proteins, which might be explained by their multiple epitope character and/or to conformational changes of the aggregated protein molecules [14,15,17]. The protein aggregation either reveals new epitopes recognized as non-self or leads to the spacing of the epitopes known to break self-tolerance [4]. Therefore, the protein aggregates should be prevented during the nanoparticle preparation steps.

In this study, for stabilizing proteins without protein aggregation and bioactivity loss, a novel approach to prepare protein-loaded nanoparticles was developed. The model proteins, granulocyte-macrophage colony-stimulating factor (GM-CSF), granulocyte colony-stimulating factor (G-CSF), *β*-galactosidase, myoglobin (MYO), and bovine serum albumin (BSA) were encapsulated into the dextran nanoparticle by aqueous-aqueous freezing-induced phase separation without contacting the aqueous/organic interface. This novel dextran nanoparticle attenuated the acidic microenvironment in the poly (lactic-co-glycolic acid) (PLGA) microsphere by means of a dilution effect and preserved protein's bioactivity during the preparation process.

## Methods

### Materials

The BSA and GM-CSF were purchased from Invitrogen Co Ltd, Shanghai, China. The dextran (mol wt., 64-76 KD), polyethylene glycol 8000, and *β*-galactosidase were obtained from Sigma (St. Louis, MO, USA). TF-1 cell line was obtained from the Chinese Institution for the Control of Pharmaceutical and Biological Products (Tiantan Xi Li, Beijing, China). The Roswell Park Memorial Institute (PRMI 1640) medium was purchased from Gibco (Life Technologies Corporation, Grand Island, NY, USA). Sodium dihydrogen phosphate, sodium chloride, sucrose, and other chemicals were purchased from the Chinese Medicine Group Chemical Reagent Corporation (Shanghai, China). Micro bicinchoninic acid (Micro BCA) protein kit was purchased from Pierce Biotechnology, Inc. (Vallejo, CA, USA).

### Preparation of dextran nanoparticles loaded with proteins

The model proteins, BSA, GM-CSF, *β*-galactosidase, and MYO were encapsulated into dextran nanoparticles according to aqueous-aqueous freezing-induced phase separation methods. Briefly, proteins were dissolved in 6% (*w/w*) dextran solutions as separated phase, and the polyethylene glycol (PEG) was dissolved to get an aqueous solution with a concentration of 6% (*w/w*). Then, the two solutions were gently mixed to get a clear solution. The solution was frozen at −80°C in the refrigerator for more than 10 h and then dried at a vacuum level below 0.1 mbar for 24 h. After lyophilization, the powder was washed with dichloromethane and subsequently centrifuged at 12,000 rpm for 3 min and three times to remove the continuous phase. Once dichloromethane was evaporated, fine dextran nanoparticles loaded with proteins were obtained.

### Morphology of dextran nanoparticles loaded with proteins

The morphology analysis was measured by scanning electron microscopy (SEM). The dextran nanoparticles were attached to a metal stub using a double-sided adhesive and exposed to gold spray under argon atmosphere for 10 min. The size distribution of dextran nanoparticles was measured using a photon correlation spectrometer (PCS) (Brookhaven, BI-90 plus, Holtsville, NY, USA). A 10-mg dextran nanoparticle was dispersed in 5 ml of isopropyl alcohol and used for PCS analysis.

### Encapsulation efficiency of proteins and recovery of dextran nanoparticle

The encapsulation efficiency of dextran nanoparticles was determined as follows: the amount of BSA, GM-CSF, and MYO recovered from the dextran nanoparticle was determined by the Micro BCA kit. The dextran nanoparticles loaded with proteins obtained were weighed and then dissolved in deionized water for Micro BCA determination. All measurements were performed in triplicate. The encapsulation efficiency of protein and recovery of the dextran nanoparticle were calculated as follows:

(1) $$\begin{array}{l} \mathrm{Encapsulation}\;\mathrm{Efficiency}\;\left(\mathrm{EE}\right)=\\ W\;\left(\mathrm{Proteins}\;\mathrm{Recovered}\;\mathrm{from}\;\mathrm{Dextran}\;\mathrm{Nanoparticle}\right)/\\ \mathrm{Wt}\left(\mathrm{Proteins}\;\mathrm{of}\;\mathrm{Theoretical}\;\mathrm{Content}\;\mathrm{in}\;\mathrm{Dextran}\;\mathrm{Nanoparticle}\right)\\ \;\times 100\% \end{array}$$

(2) $$\begin{array}{l} \mathrm{Recovery}\;\left(\%\right)=\\ W\;\left(\mathrm{Dextran}\;\mathrm{Nanoparticle}\;\mathrm{Collected}\right)/\\ \mathrm{Wt}\left(\mathrm{Dextran}\;\mathrm{Nanoparticle}\;\mathrm{of}\;\mathrm{Theoretical}\;\mathrm{Content}\right)\times 100\% \end{array}$$

### Assay of protein aggregation

The BSA, GM-CSF, and G-CSF were selected as model proteins to examine the protein aggregation during the preparation process. The size-exclusion chromatography-high- performance chromatography (SEC-HPLC) was used to identify proteins and analyze the monomer protein content recovered. SEC-HPLC provides information on the size of the proteins and the presence of aggregated proteins. The extracted proteins from samples were determined using a TSK G2000 Sk-XL1 column (YL Instrument, Co., Anyang, Korea). An isotonic phosphate buffer (25 mM sodium phosphate, 100 mM NaCl; pH = 7.4) was used as mobile phase at a flow rate of 1.0 ml/min. The examination was carried out by UV monitoring at 214 nm. The BSA, GM-CSF, and G-CSF were also dissolved in distilled water and then dispersed in dichloromethane to get controlled water-in-oil (W/O) emulsion. The controlled emulsion and standard protein solutions were also subject to SEC-HPLC for comparing with dextran nanoparticles loaded with proteins.

### Bioactivity assay of proteins during the formulation steps

The GM-CSF, G-CSF, and *β*-galactosidase were selected as model proteins to examine the bioactivity during the process. The bioactivity of the GM-CSF recovered during the steps was determined by the proliferation effect induced on TF-1 cell line. The TF-1 cells were grown in a PRMI 1640 medium supplemented with 10% fetal bovine serum (FBS). The cultures were maintained in plastic flasks and incubated in CO_2_/air (5:95, *v/v*) at 37°C in a humidified incubator. The bioactivity of the G-CSF recovered was determined by the proliferation effect induced on an NSF-60 cell line. The NFS-60 cells were grown in a PRMI 1640 medium supplemented with 10% FBS. The cultures were maintained in plastic flasks and incubated in CO_2_/air (5:95, *v/v*) at 37°C in a humidified incubator. The catalysis bioactivity of the *β*-galactosidase on o-nitrophenol recovered was determined by the ortho-nitrophenyl-*β*-galactoside (ONPG) assay. The assay was carried out according to a protocol from Sigma. Protein activity was determined by the absorbance of the reaction product of ONPG at 420 nm. The *β*-galactosidase and GM-CSF were also dissolved in distilled water and then were dispersed in dichloromethane to get the controlled W/O emulsion. The controlled emulsion and standard protein solutions were also subject to bioactivity assay for comparing with dextran nanoparticles loaded with proteins.

### Ability of dextran nanoparticle to overcome acidic microenvironment

LysoSensor™ Yellow/Blue dextran (Life Technologies Corporation, Grand Island, NY, USA) was loaded into the dextran nanoparticle to evaluate the ability to attenuate the local acidic microenvironment in the PLGA microsphere during the *in vitro* release period. The dextran nanoparticles were encapsulated into composite PLGA microsphere by the solid-in-oil-in-water method [15]. Accordingly, the LysoSensor™ Yellow/Blue dextran solution was encapsulated into the PLGA matrix to act as the controlled sample by the traditional water-in-oil-in-water (W/O/W) double emulsion method [9]. To monitor the change in pH within PLGA microspheres vs. time, 10 mg of dried PLGA microspheres loaded with the LysoSensor™ Yellow/Blue dextran were incubated in tubes containing 1 ml of 20-mM PBS buffer at 37°C under 90 rpm continuously for 12 days. At pre-determined time intervals, the supernatant was removed and then replenished with PBS to vortex to re-suspend the microspheres. The ratio imaging was conducted on fluorescent microscope (Olympus, IX71-32PH, Shinjuku-ku, Tokyo, Japan). The PLGA microsphere was excited at 335 and 381 nm, and the images emitted at 452 and 521 nm were taken for analysis. The fluorescent intensity was analyzed using the software, WASABI V.1.4. The standard curve of ratio of fluorescent intensity vs. pH was generated by placing the LysoSensor™ Yellow/Blue dextran-loaded dextran nanoparticles at a known pH on a microscope slide. Multiple images were taken at each pH and then averaged to obtain the standard curve.

## Results and discussion

### Morphology of dextran nanoparticle

**Figure 1:**
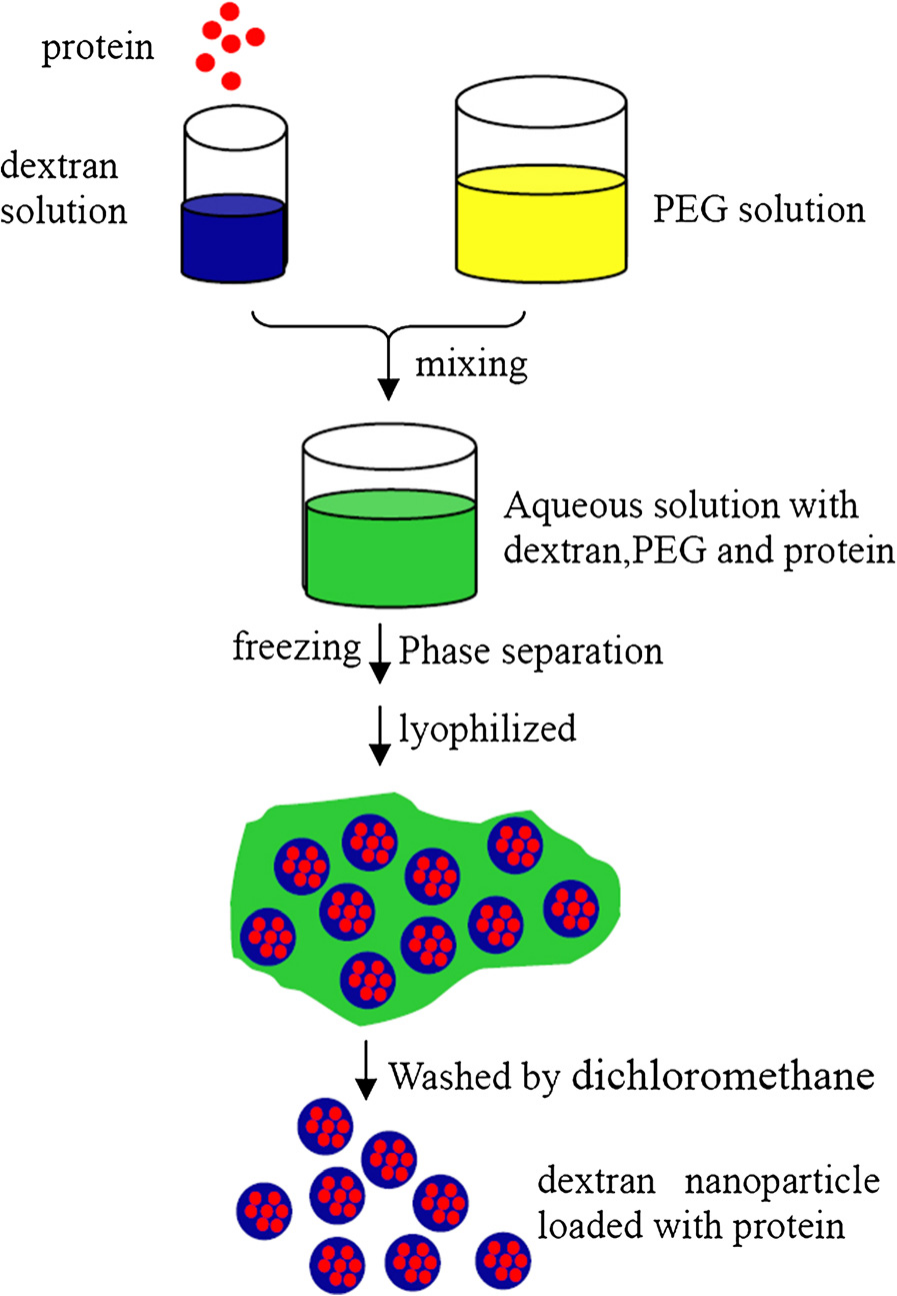
The formulation strategy of fabricating the dextran nanoparticles loaded with proteins.

The strategy for fabricating dextran nanoparticles loaded with proteins is shown in Figure 1. Briefly, proteins and PEG were dissolved in dextran solutions and aqueous solution, respectively. After these two solutions were mixed to get a clear solution, the solution was frozen dried under vacuum and washed with dichloromethane to give fine dextran nanoparticles loaded with proteins.

**Figure 2:**
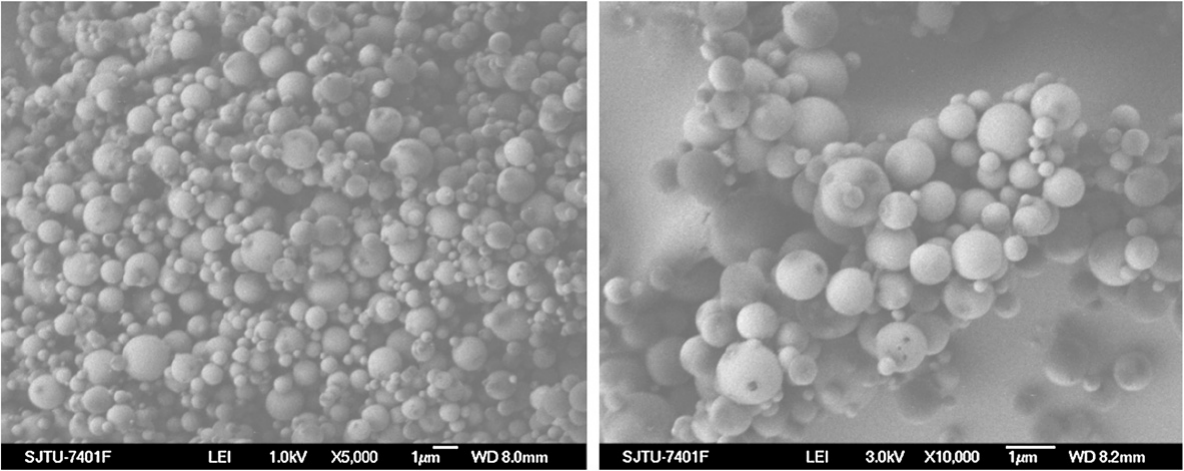
An SEM photo of dextran nanoparticles loaded with BSA.

**Figure 3:**
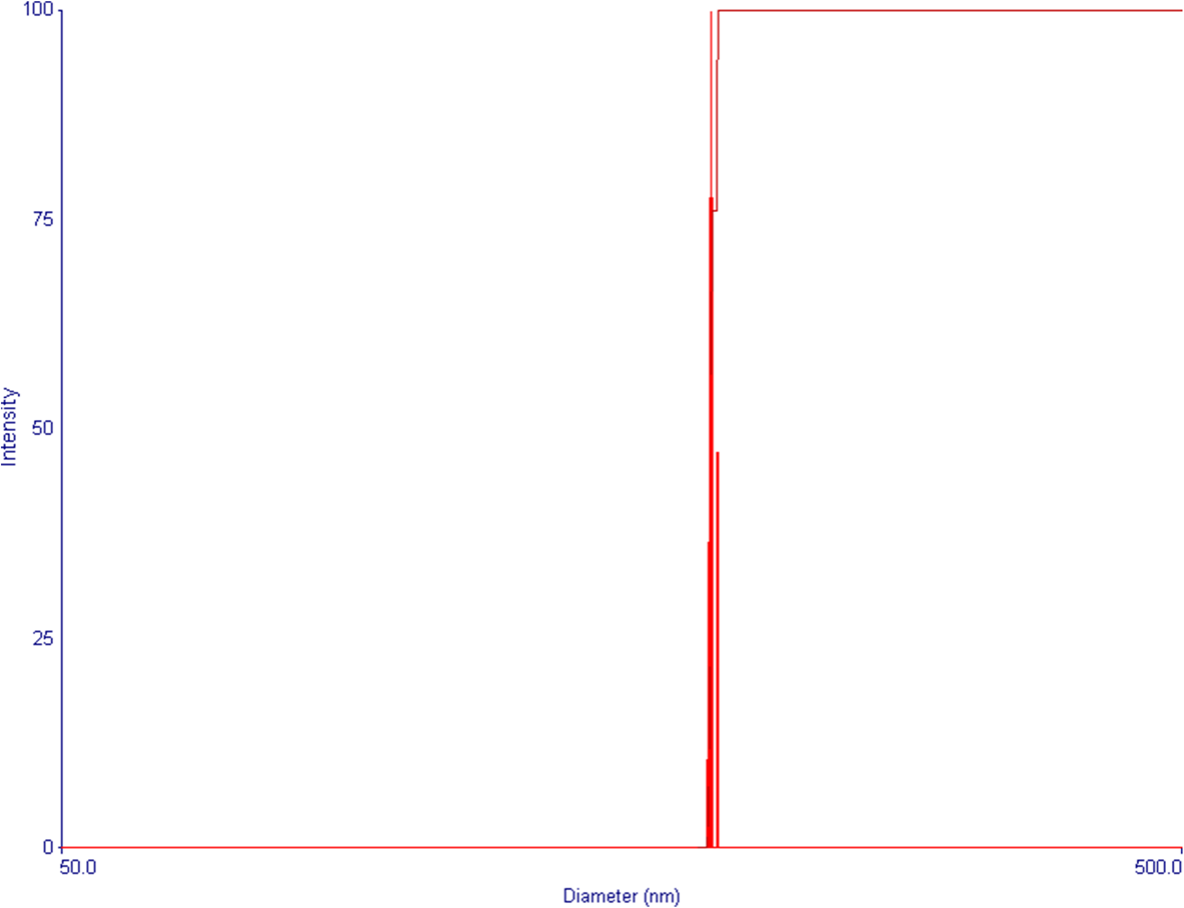
The size distribution of dextran nanoparticles loaded with BSA.

Figure 2 shows SEM images of dextran nanoparticles loaded with BSA (DP-BSA). DP-BSA exhibit a spherical shape, smooth surfaces, and diameters ranging from 200 to 500 nm. These results are consistent with that of the particle size analysis which shows the effective diameter of 293 nm for DP-BSA (Figure 3).

### Encapsulation efficiency of dextran nanoparticles

As shown in Table 1, the encapsulation efficiency of dextran nanoparticles loaded with different proteins was generally larger than 98%. The recovery of proteins extracted from dextran nanoparticles ranged from 65% to 72%. Some proteins might be washed away by dichloromethane during the preparation process.

**Table 1:** **The encapsulation efficiency and recovery of dextran nanoparticles (*n *= 3)**

**Number**	**Protein**	**Encapsulation efficiency(ave% ± SD)**	**Recovery (%) (ave% ± SD)**
1	BSA	99.23 ± 1.69	71.26 ± 2.06
2	GM-CSF	98.37 ± 1.27	69.16 ± 2.78
3	MYO	98.16 ± 1.55	65.57 ± 1.56

### Protein aggregation during the formulation steps

**Figure 4:**
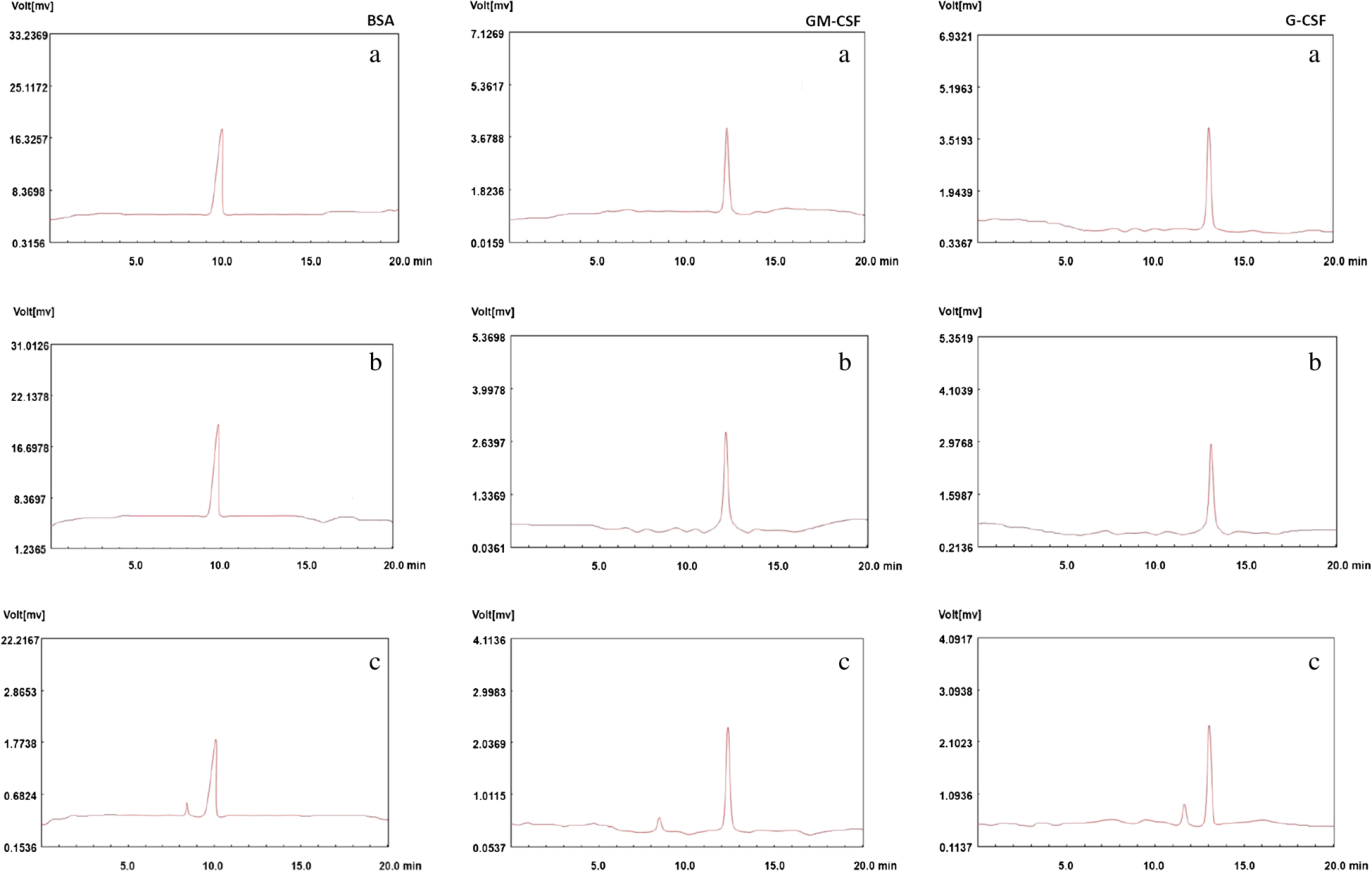
SEC-HPLC of model proteins recovered from standard solution (a), dextran nanoparticle (b), and W/O emulsion (c).

In order to address this novel dextran nanoparticle that may protect proteins from aggregation during the formulation process, the BSA, GM-CSF, and G-CSF were selected as model proteins, and SEC-HPLC was used to characterize the protein extracted from the protein standard solution, dextran nanoparticle, and controlled W/O emulsion. Figure 4 shows the SEC-HPLC charts of BSA extracted from the BSA standard solution, dextran nanoparticle, and W/O emulsion. The peak of BSA samples around 9.8 and 8.2 min were ascribed to the monomer and dimer BSAs, respectively. As shown in Figure 4, only one peak corresponding to the monomer BSA was observed in the BSA solution and dextran nanoparticle. However, two peaks corresponding to the monomer and dimer BSAs were observed in the controlled W/O emulsion. We found similar results in the GM-CSF and G-CSF samples, as shown in Figure 4. Only monomer GM-CSF (or G-CSF) was extracted from the dextran nanoparticle, exactly the same as those from protein standard solutions, whereas dimer GM-CSF (or G-CSF) can be observed in the controlled W/O emulsion. This result indicated that the encapsulation of model proteins into the dextran nanoparticle did not cause protein aggregation during the preparation step.

### Bioactivity of proteins during the formulation steps

**Figure 5:**
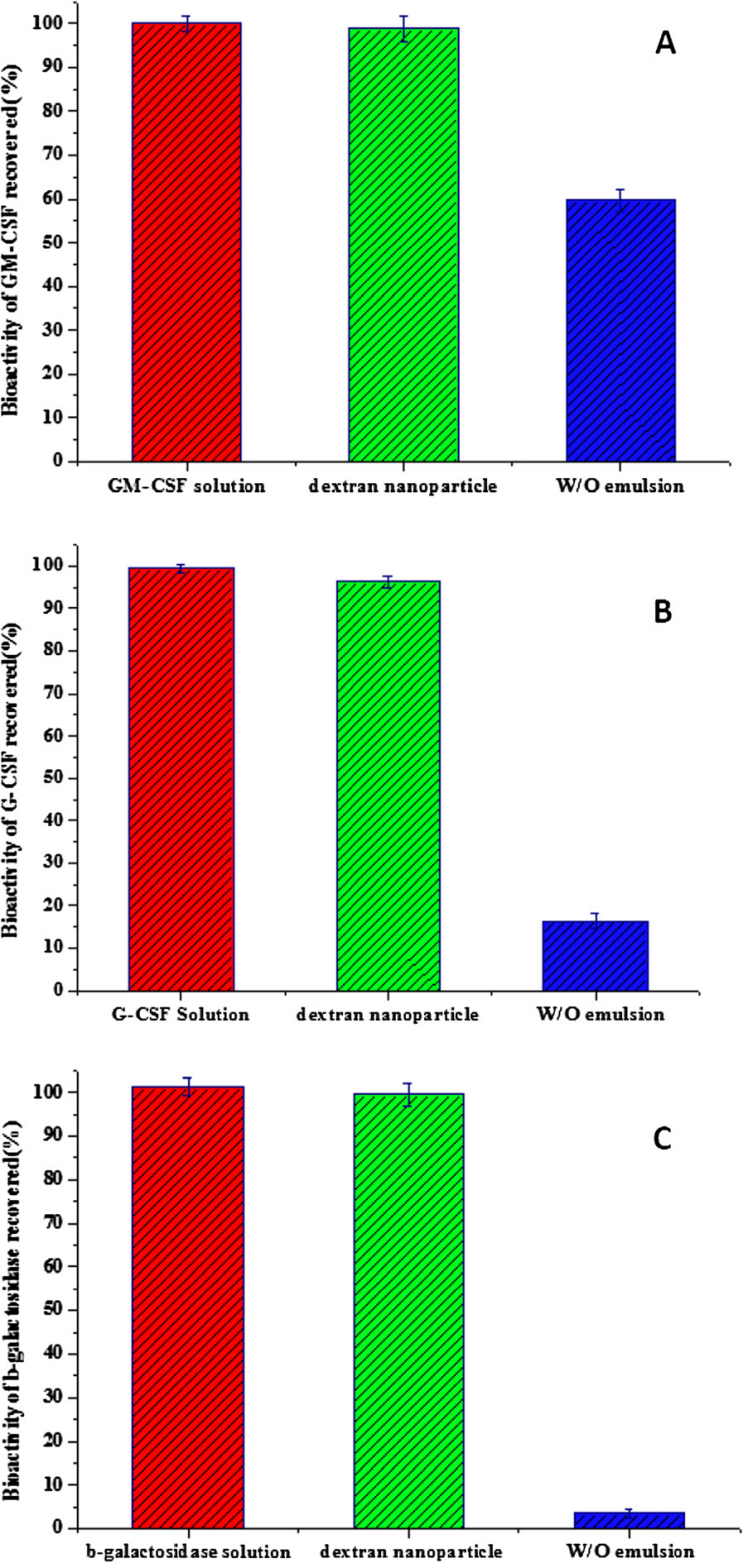
**Bioactivity of model proteins recovered from standard solution, dextran nanoparticle, and W/O emulsion.** GM-CSF (**A**), G-CSF (**B**), *β*-galactosidase (**C**).

In order to address this novel dextran nanoparticle that may protect proteins from bioactivity loss during the formulation process, the proliferative abilities of TF-1 and NFS-60 cell line were measured to assess the bioactivity of GM-CSF (Figure 5A), G-CSF (Figure 5B), and *β*-galactosidase (Figure 5C) which were recovered from the protein standard solution, dextran nanoparticle, and controlled W/O emulsion. The results indicate that the proteins recovered from the dextran nanoparticle retained same bioactivity as those recovered from protein standard solution, and show much higher bioactivity than those recovered from controlled W/O emulsion. These results further confirmed that proteins could be well stabilized after they were encapsulated into the dextran nanoparticle.

### Ability of dextran nanoparticle to overcome acidic microenvironment

**Figure 6:**
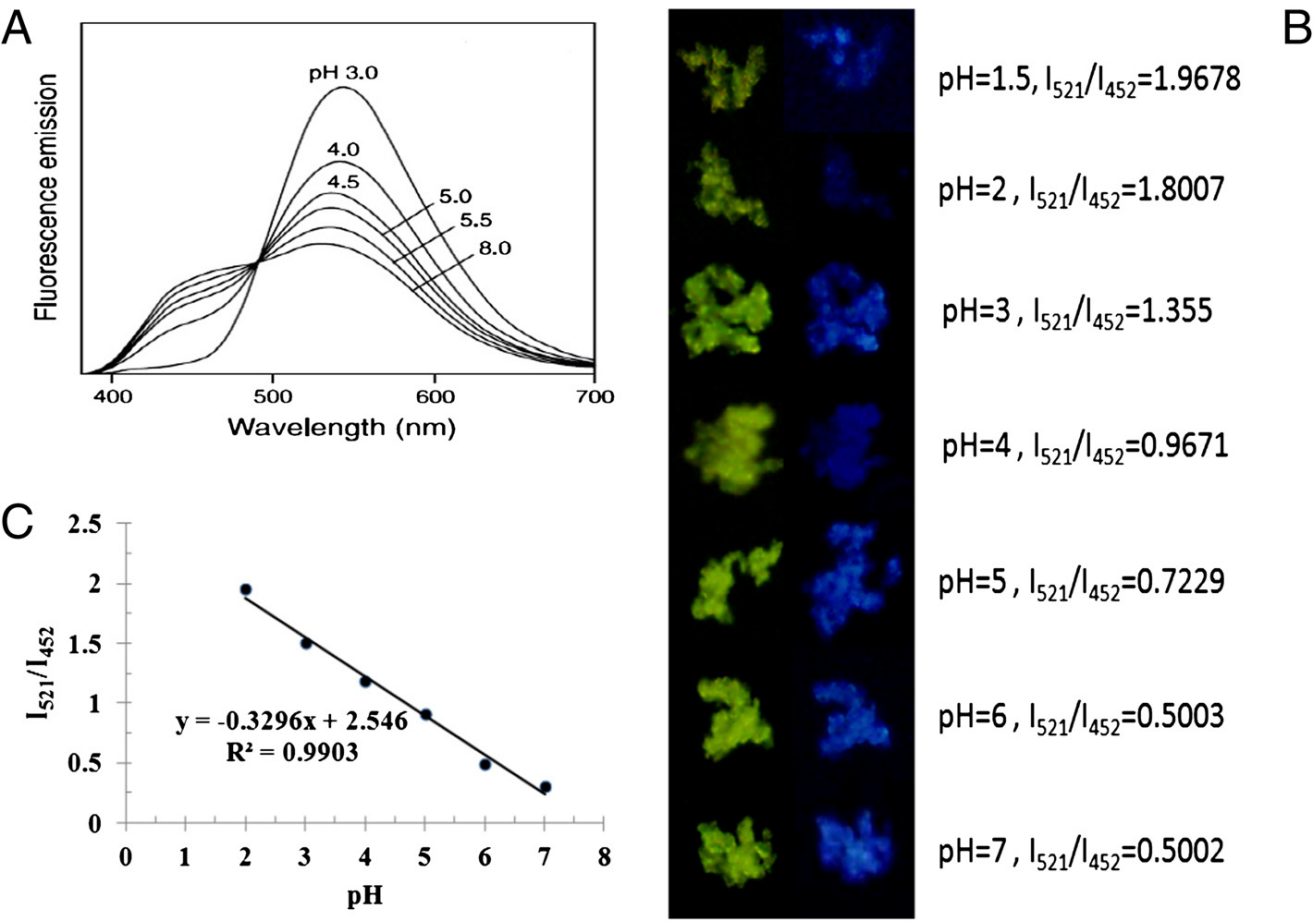
**The relation of fluorescent intensity ratio and pH.** Assay mechanism (**A**), standard curve of fluorescent intensity ratios of the LysoSensor™ Yellow/Blue dextran vs. pH (**B**), fluorescence image of dextran nanoparticle taken at λem = 521,452 nm (**C**).

Generally, the pH has been shown to affect the stability of proteins. At an acidic microenvironment, many proteins tend to unfold to aggregate. Therefore, many studies have been developed to overcome the acidic microenvironment around the protein and stabilize proteins during the *in vitro* release period. In order to evaluate the ability of dextran nanoparticle to attenuate the acidic microenvironment, the dextran nanoparticle was encapsulated into PLGA microspheres in which acidic microenvironment can be produced via biodegradation of PLGA. The LysoSensor™ Yellow/Blue, a fluorescent anisotropic probe, was used to label and track acidic organelles. Figure 6 described the relationship between fluorescent intensity ratio and the pH value. It can be seen that the fluorescent intensity ratio at 452 and 521 nm of the LysoSensor™ Yellow/Blue loaded in the dextran nanoparticle linearly correlates with the pH in the range from 2.0 to 7.0.

**Figure 7:**
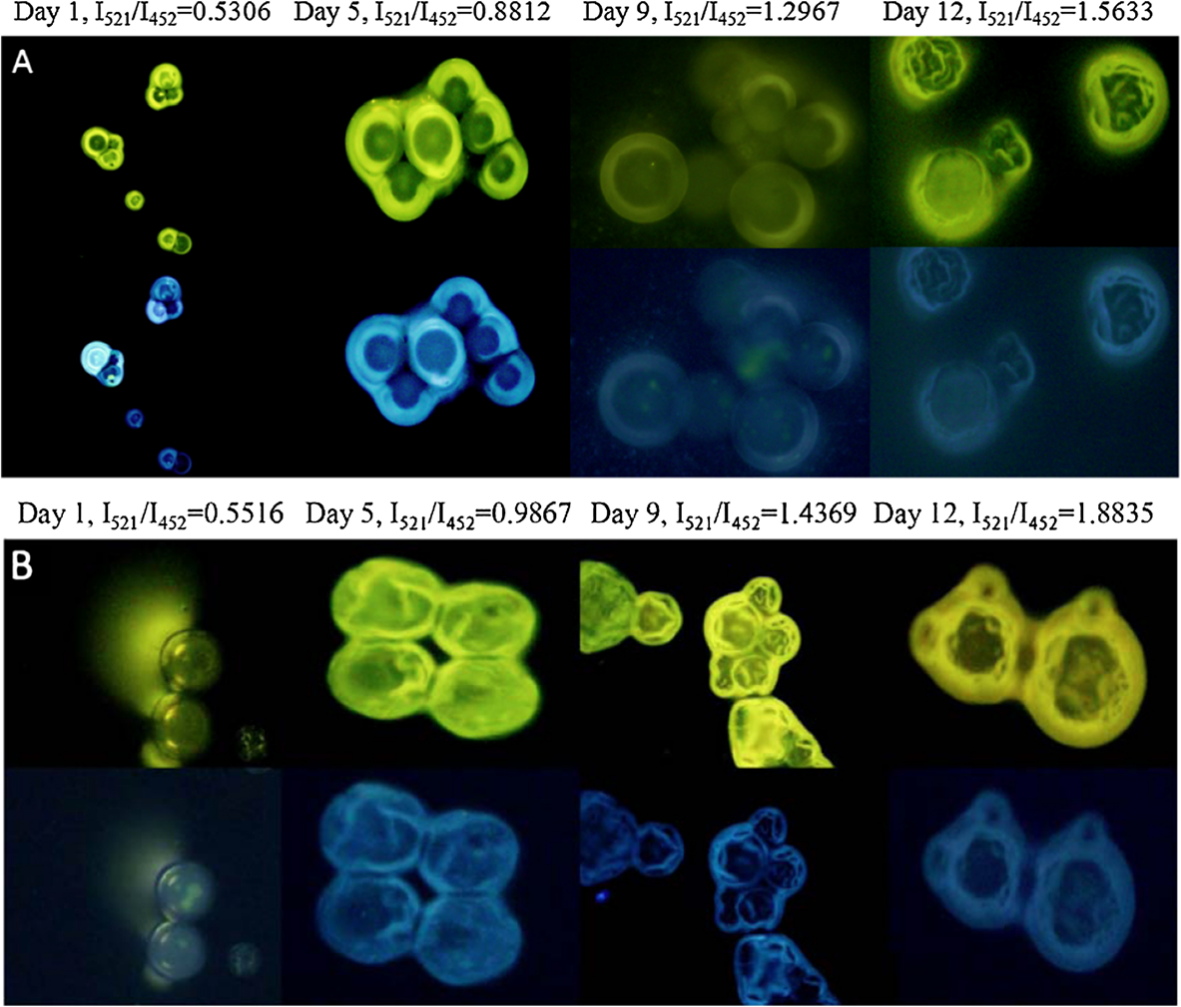
**Fluorescent image of LysoSensor™ Yellow/Blue dextran-loaded PLGA microspheres.** λem = 521,452 nm during the *in vitro* release period. Dextran nanoparticles loaded in PLGA microsphere (**A**), the controlled LysoSensor™ Yellow/Blue dextran solution loaded in PLGA microsphere by traditional W/O/W method (**B**).

Figure 7A,B showed the fluorescent images of microspheres loaded with the LysoSensor™ At days 1,5,9, and 12, the fluorescent intensity ratio emitted at 521 and 452 nm from the dextran nanoparticle increased from 0.5306, 0.8812, and 1.2967 to 1.5633, corresponding to a pH decrease from 6.11, 5.05, and 3.79 to 2.98. Accordingly, at days 1,5,9, and 12, the of fluorescent intensity ratio emitted at 521 and 452 nm from the LysoSensor™ Yellow/Blue dextran solution entrapped in the PLGA microsphere increased from 0.5516, 0.9867, and 1.4396 to 1.8835, corresponding to a pH decrease from 6.05, 4.73, and 3.36 to 2.01. The PLGA microspheres loaded with dextran nanoparticles were swollen to a much larger extent compared to the controlled PLGA microspheres by the traditional W/O/W method. The acid caused by PLGA degradation was diluted but not neutralized in microspheres. Therefore, the acidic microenvironment in the PLGA microsphere may be attenuated by the dilution effect. It is especially preferred to improve the stability of those acid-sensitive proteins.

## Conclusion

This present study developed a novel approach to prepare dextran nanoparticles to stabilize and encapsulate proteins. The BSA, GM-CSF, MYO, and *β*-galactosidase were selected as model proteins to characterize the dextran nanoparticles. The proteins were successfully encapsulated into the dextran nanoparticle with spherical morphology, suitable particle size, and high encapsulation efficiency. There were no protein aggregation and bioactivity loss during the formulation steps. The dextran nanoparticles also improved the stability of acid-sensitive proteins. This unique method may provide a promising way to stabilize proteins.

## Competing interests

The authors declare that they have no competing interests.

## Authors’ contributions

FW performed the experiment. LMW and WEY designed the experiments and wrote the manuscript. JS participated in the measurements. ZHZ and TJ provided useful suggestions. All authors read and approved the final manuscript.
